# The ISUP system of staging, grading and classification of renal cell neoplasia

**DOI:** 10.15586/jkcvhl.2014.11

**Published:** 2014-07-20

**Authors:** Hemamali Samaratunga, Troy Gianduzzo, Brett Delahunt

**Affiliations:** 1 Aquesta Pathology, Brisbane, Queensland, Australia; 2 University of Queensland, Brisbane, Queensland, Australia; 3 Wesley Hospital, Brisbane, Queensland, Australia; 4 Department of Pathology and Molecular Medicine, Wellington School of Medicine and Health Sciences, University of Otago, Wellington, New Zealand

## Abstract

There have been significant changes in the staging, classification and grading of renal cell neoplasia in recent times. Major changes have occurred in our understanding of extra-renal extension by renal cell cancer and how gross specimens must be handled to optimally display extra-renal spread. Since the 1981 World Health Organization (WHO) classification of renal tumors, in which only a handful of different entities were reported, many new morphological types have been described in the literature, resulting in 50 different entities reported in the 2004 WHO classification. Since 2004, further new entities have been recognized and reported necessitating an update of the renal tumor classification. There have also been numerous grading systems for renal cell carcinoma with Fuhrman grading, the most widely used system. In recent times, the prognostic value and the applicability of the Fuhrman grading system in practice has been shown to be, at best, suboptimal. To address these issues and to recommend reporting guidelines, the International Society of Urological Pathology (ISUP) undertook a review of adult renal neoplasia through an international consensus conference in Vancouver in 2012. The conduct of the conference was based upon evidence from the literature and the current practice amongst recognized experts in the field. Working groups selected to deal with key topics evaluated current data and identified points of controversy. A pre-meeting survey of the ISUP membership was followed by the consensus conference at which a formal ballot was taken on each key issue. A 65% majority vote was taken as consensus. This review summarizes the outcome and recommendations of this conference with regards to staging, classification and grading of renal cell neoplasia.

## Introduction

Renal cancer is one of the most common visceral malignancies with a significant rate of cancer related deaths in both males and females (1, 2). While surgical removal is the gold standard treatment for localized kidney cancer, many targeted therapies have been recently introduced for the treatment of metastatic renal cell cancer (3–5). Accurate diagnosis, grading and staging are crucial in the management of these patients, both to improve outcome as well as to allow for accurate prognostication. Staging of renal cell cancer is one of the most important predictors of prognosis and among staging criteria it has recently been recognized that renal sinus invasion is the most common route of extra-renal spread (6, 7). Bonsib et al. (8) showed > 90% of clear cell renal cell carcinoma (RCC) ≥ 7cm in diameter to have invaded the renal sinus. In a further study, Thompson et al. (9) examined additional tissue from nephrectomy specimens from patients with pT1 disease who had died of RCC. Renal sinus invasion was found in 14 (42%) of these cases and they compared these findings with a matched set of 33 patients who had not died of RCC. In this latter group renal sinus invasion was seen in only 2 (6%) of cases. Appropriate sampling of a kidney tumor, including adequate sampling of the renal sinus has been shown to be extremely important for the correct staging of kidney tumors (8, 10). In addition to recognizing this, the 7^th^ edition of the TNM staging system of the International Union Against Cancer/American Joint Commission on Cancer/ (UICC/AJCC) introduced several changes to the staging system of RCC (11, 12). In parallel with this renal tumor classification has undergone major changes in the last three decades, with novel morphotypes being added to successive classification systems (13–15). Also during this time several grading systems for RCC have been proposed, of which the Fuhrman grading system had achieved most popularity (16). In recent years, the value of this system has been questioned, not only with regards to its applicability in practice, but also with regards to its value as a prognostic marker (17–19).

In order to address these issues the International Society of Urological pathology (ISUP), convened a consensus conference to produce guidelines and made recommendations regarding the handling/sampling and staging, classification and grading, of adult kidney RCC based on current practice and evidence from the literature (20–24). Here we present a summary the results of this ISUP consensus meeting.

## Handling and staging of RCC

Appropriate handling is clearly the first step toward accurate diagnosis and staging of RCC and these issues were considered by an expert group convened prior to the conference. After deliberation by the conference delegates, it was recommended that the initial sampling section should be along the long axis of the kidney. It was considered that this could be in the mid lateral plane of the kidney or through the collecting system or vascular system. It was agreed that the optimal method to identify renal sinus venous invasion is to open the kidney along probes placed in large venous channels. It was further agreed that margin involvement should be assessed by inking suspicious areas, the perinephric fat margin and hilum of radical nephrectomy specimens or the renal parenchymal resection margin and perinephric margin of partial nephrectomy specimens. At present there is no information in the literature as to how best demonstrate perinephric fat invasion and it was agreed that the fat overlying the tumor should be kept intact, with multiple perpendicular cuts made, with a view to sampling suspicious areas.

## Tumor measurements

Tumor size is an important determinant of the UICC/AJCC TNM pathologic stage (11, 12) and correlates with perinephric fat extension, renal sinus invasion, prognosis and metastatic potential. There are several confounding factors in the estimation of tumor size. Retrograde venous invasion, renal vein and vena cava tumor, which can invade through the venous wall to achieve confluence, can cause problems with tumor size measurements. There is currently no guidance in the literature as to how size should be measured and whether or not a renal vein thrombus should be included in the measurement of the main tumor mass. It was agreed that after bivalving the kidney, multiple further sections (usually perpendicular) should be examined to ascertain the maximum tumor dimension. Although areas of contiguous involvement of perinephric fat and renal sinus tissue should be included in the tumor measurement, satellite nodules should not. There was consensus that the tumor measurements should not include a renal vein or caval thrombus. In cases with multiple tumors, there was near consensus (survey 62%) that all tumors up to some designated maximum (eg.5) should be measured, with fewer participants in favour of providing a measurement for the largest two tumors, as well as a range for all other tumors present.

## Gross examination for lymph nodes

While there has been a suggestion that both peri-renal and hilar fat should be dissected in order to detect lymph nodes (25), it has also been claimed that palpation and dissection of the renal hilar area only is sufficient (26). Mehta et al. (27) examined the entire hilar tissue for histology and found that 80% of grossly visible lymph nodes were positive for tumor, whereas microscopic lymph nodes were all benign.

In view of this it would appear that examining the grossly evident lymph nodes only is sufficient and there was consensus that dissection of the hilar fat, for the purpose of identifying lymph nodes, is sufficient.

## Sampling tumor

Previous sampling recommendations have been to take at least one block/cm of greatest dimension of tumor (26, 28). Areas of different appearance or consistency and blocks to demonstrate tumor relationship with peri-renal fat, renal sinus, renal pelvis and adrenal gland should also be taken. There was consensus that sampling should follow this general guideline of sampling with a minimum of three blocks. Multiple tumors are commonly found in hereditary syndromes such as von Hippel-Lindau disease, hereditary papillary RCC, Tuberous sclerosis and Birt-Hogg-Dube syndrome (29–31). Multiple tumors also occur with oncocytosis, acquired cystic kidney disease and with papillary RCC (32, 33). Multifocality in sporadic RCC is rare. It is extremely uncommon to have >5 tumors in one kidney and in such instances sampling issues are not addressed in the literature. There was consensus that in cases with multiple tumors, sampling should include the 5 largest tumors at a minimum.

## Sampling the renal sinus

The renal sinus is the fatty tissue compartment that lies between renal parenchyma and the pelvi-calyceal system. This is a complex structure with the vascular system being anterior to the pelvi-calyceal system and the sinus extending anteriorly and posteriorly within the kidney (7). Veins entering the renal sinus have a smooth muscle media of variable thickness. Gross recognition of renal sinus involvement is often not difficult and rounded nodules in the renal sinus outside the main tumor indicate sinus vein invasion (Fig. 1A). Sinus fat invasion occurs when intravenous tumor invades through the media (33), however, in some cases tumor bulging into the sinus can be difficult to interpret. Recommendations for sampling have varied with protocols ranging from sampling of the entire interface to 2–3 blocks (6, 7, 34, 35). At the meeting there was consensus that when invasion of the renal sinus is uncertain, at least three blocks of the interface should be taken. If invasion is grossly evident or obviously not present (small peripheral tumor) only 1 block is needed to confirm the gross impression.

**Figure 1A.**
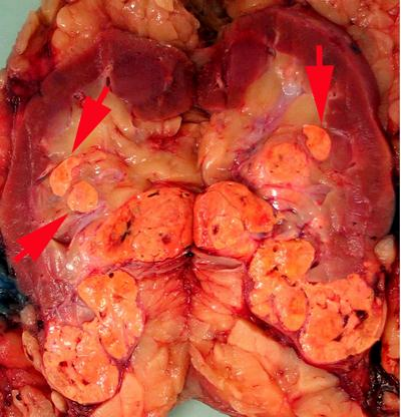
Rounded nodules in the renal sinus indicate sinus vascular involvement.

**Figure 1B.**
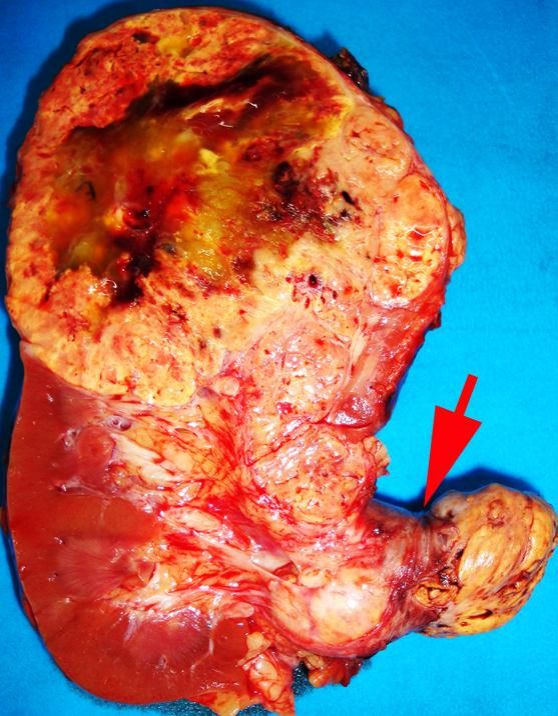
Tumor grossly involving the renal vein is usually visible to the naked eye. A tumor thrombus typically expands the renal vein. The renal vein margin (arrow) often retracts back from the tumor thrombus when vein clamps are removed.

## Sampling renal vein and vena cava

Tumor grossly involving the renal vein is usually visible to the naked eye. A tumor thrombus typically expands the renal vein (Fig. 1B) and may or may not be adherent to the renal vein wall. If the surgical margin is clamped, there is a tendency for the renal vein margin to retract back from the tumor thrombus when the clamps are removed. There was near consensus that the actual margin and additional sections of the tumor thrombus should be sampled, particularly in areas where tumor is adherent to the wall.

Tumor invading into the vena cava wall has prognostic significance (35). In the 7^th^ edition of the UICC/AJCC staging classification, tumor extending into the vena cava above the diaphragm or invading vena cava wall is classified as pT3c. In cases where a caval thrombus is present below the diaphragm, identification of invasion of the caval wall alters the staging category from pT3b to pT3c (12), thus impacting adversely on prognosis. It is therefore mandatory that adequate sections be examined, so as not to overlook this. When a specimen is submitted separately as a “caval thrombus” there was consensus that two or more sections must be taken to look for presence of tumor invading the caval wall.

## Sampling of normal renal parenchyma

Since a kidney removed for neoplastic disease may also have concurrent non-neoplastic renal pathology including glomerular, tubulointerstitial and vascular disease, it was recommended that normal parenchyma with tumor, and normal parenchyma distant from the tumor be sampled.

## Renal sinus invasion

In the renal sinus, large veins are thought to become involved before small veins and as such small vein involvement usually implies large vein involvement. The renal sinus has rich venous anastomoses, therefore any venous involvement is likely to have metastatic risk. The majority of participants in the pre-meeting survey agreed that contact with renal sinus fat, (Fig. 2A) or loose connective tissue, clearly beyond the renal parenchyma indicated renal sinus invasion. Large sinus veins, including segmental branches of renal vein have marked variability in amount of smooth muscle and it was agreed that, if there are any endothelial lined spaces containing tumor within the renal sinus, regardless of size, this must also be considered renal sinus invasion (pT3a) (Fig. 2B).

**Figure 2A.**
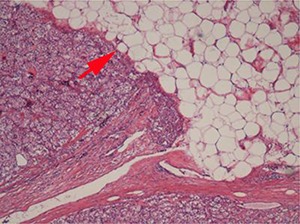
Carcinoma in contact with renal sinus fat indicates renal sinus invasion.

**Figure 2B.**
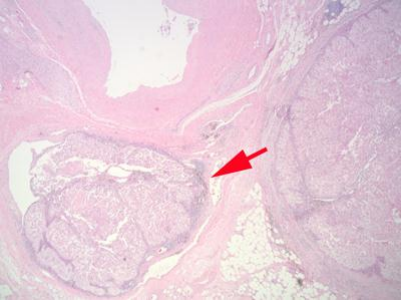
Carcinoma within vascular channels within the renal sinus, regardless of size is also considered renal sinus invasion.

## Perinephric fat invasion

Perinephric fat is present outside the renal capsule within the confines of the Gerota fascia. Since many RCC arise in the renal cortex they may protrude into the perinephric fat, distorting the renal contour. The presence of a smooth, convex outer surface, even if tumor protrudes well into the perinephric fat, does not constitute perinephric fat invasion (Fig. 3A). When RCC invades through the renal capsule into perinephric fat, there is loss of the smooth convex outer contour, with nodules or irregular tumor masses protruding into the perinephric fat (Fig. 3B). Microscopically, perinephric fat invasion is confirmed when there is tumor touching fat or extending as irregular tongues into perinephric tissues, with or without desmoplasia (Fig. 3C).

**Figure 3A.**
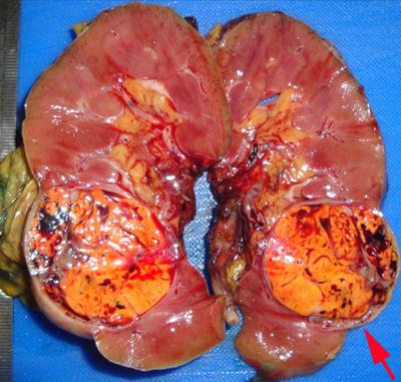
If the outer surface of the tumor is smooth and convex, even if tumour protrudes well into the perinephric fat, it is not considered perinephric invasion.

**Figure 3B.**
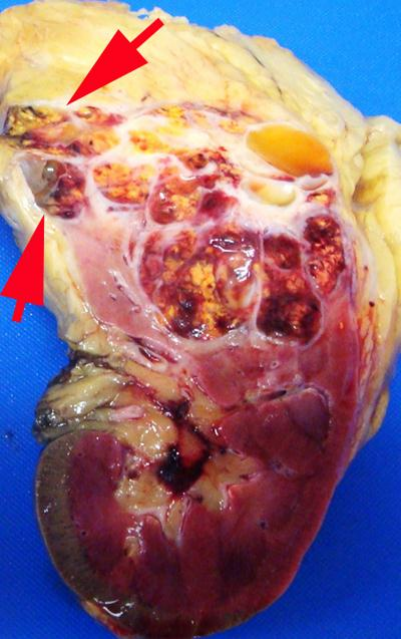
When RCC invades through the renal capsule into the perinephric fat, there is loss of the smooth convex outer contour, with nodules or irregular tumor masses protruding into the perinephric fat.

**Figure 3C.**
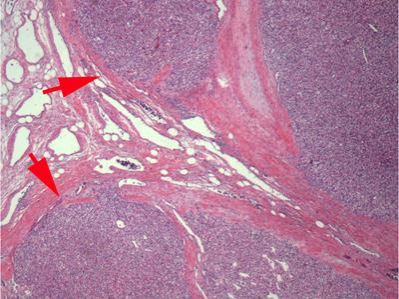
Tumor extending as irregular tongues into perinephric tissues with or without desmoplasia is considered perinephric fat invasion.

## Venous invasion

There was agreement amongst participants in the survey that, to diagnose renal vein margin positivity, there must be microscopically confirmed adherent tumor at the actual margin. If there is microscopic infiltration of the inferior vena cava by tumor, this is considered to be caval involvement. Tumor in vascular channels of any size in the renal sinus, including segmental branches of renal vein, must be reported as this is considered indicative of pT3a staging category.

## Adrenal gland involvement

**Figure 4.**
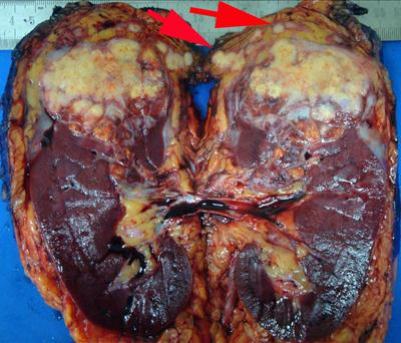
When the adrenal gland is involved it must be established whether it is direct invasion (pT4) or metastatic spread (pM1). In this example, both appear to be present.

In the current UICC/AJCC TNM staging classification (11) direct invasion of the adrenal gland is considered to be pT4 disease. This is a significant change from the 2002 UICC/AJCC TNM classification, in which adrenal gland invasion was considered to be pT3 disease. This change is based on reports that adrenal gland involvement has a significantly worse prognosis than that of perinephric fat invasion (36, 37). In view of this the adrenal gland should be carefully examined to assess whether it is involved by carcinoma, and if so, whether this is by direct invasion (pT4) or metastatic spread needs to be assessed (Fig. 4).

## Renal tumor classification (ISUP Vancouver classification of renal neoplasia)

There were a number of recommendations with regards to modifications to the 2004 World Health Organization (WHO) classification of renal tumors. Five distinct and novel epithelial malignancies were added to the classification. These are tubulocystic RCC (38), acquired cystic disease-associated RCC (39), clear cell (tubulo) papillary RCC (40), microphthalmia transcription factor family (MiTF) translocation RCC (41) and hereditary leiomyomatosis RCC syndrome-associated RCC (42). There was agreement that, as reports of most of these new entities contained too few cases to enable prognostication; this should not be formulated at this time.

An exception to this was clear-cell (tubulo) papillary RCC which was considered to be a low-grade malignancy with a very favorable prognosis. The position of succinate dehydrogenase B deficiency-associated RCC (43) ALK translocation associated RCC (43, 44) and thyroid-like follicular RCC (45) in the new classification was considered. It was agreed that, while these entities was sufficiently well defined to enable identification, further reports were necessary to permit a clear understanding of the nature and behavior of these tumors. As such these tumors were considered emerging or provisional new entities and were not included in the Vancouver classification.

New concepts relating to recognized tumors included considering multicystic clear cell RCC as a neoplasm of low malignant potential as to date it has shown to have a universally favourable outcome (46). It was agreed that subtyping of papillary RCC (type 1, type 2 and other) was of value, however, oncocytic papillary RCC was not accepted as a distinctive entity in the new classification. Hybrid oncocytic chromophobe tumor (HOCT) was, for the time being, included as a subtype in the category of chromophobe RCC (47, 48). HOCT is an apparently indolent tumor that occurs in 3 distinct clinical settings: Birt- Hogg- Dube syndrome, renal oncocytosis and sporadic neoplasm.

Advances in our understanding of the behavior of angiomyolipomas, including epithelioid and cystic variants, were discussed. It was agreed that epithelioid angiomyolipoma should be classified according to presence or absence of atypia, as this would more accurately predict outcome of these tumors than the recognition of the presence of an epithelioid morphology without further qualification.

The relationship between cystic nephroma and mixed epithelial stromal tumor of kidney was discussed with a consensus that these tumors represent the same spectrum of neoplasia. Finally, synovial sarcoma was removed from the mixed epithelial and mesenchymal category and placed within the sarcoma group. The new classification agreed to at the meeting should be cited as the International Society of Urological Pathology Vancouver Classification of Renal Neoplasia.

## ISUP Grading Classification

There have been numerous grading systems for RCC, of which the Fuhrman grading system has achieved most popularity in clinical practice. (16, 49–51). Despite this, the value of this system in assessment of prognosis has been questioned (17, 19). Several studies have found that the Fuhrman grading system has prognostic significance only when the data are grouped (e.g. grade 1+ grade 2 versus grade 3+ grade 4), which effectively reduces the grading system to a 2 tier system. These results are somewhat similar to Fuhrman’s original report in which grade 2 and grade 3 tumors were found to have similar survival with combined grades 2 and 3 tumors, differing significantly in outcome from grade 1 and grade 4 tumors. This reduces the value of this system as the majority of RCC fall within the 2 central grading categories. Another problem is that Fuhrman’s validating study was based on a mixed series of RCC, which introduced an uncontrolled variable in the outcome studies based upon these data.

The application of the Fuhrman grading system in practice is complicated as there are three separate parameters within each grade, and involves the simultaneous assessment of nuclear size, nuclear shape and nucleolar prominence. There are no directions as to how these parameters should be stratified if they individually provide conflicting information. It is not surprising that this grading system has been shown to have poor to moderate inter-observer reproducibility and this is likely to be due to the subjective nature of the grading process (17). To compensate for this, many pathologists using the Fuhrman grading system have utilized nucleolar grade alone, which is not the recommendation of the Fuhrman grading system.

Recent studies have shown that nucleolar grade alone is sufficient to define grades 1 to 3 for clear cell and papillary RCC and that this provides outcome prediction superior to that of Fuhrman grading. In these studies, grade was based upon the single high power field showing the highest grade (52, 53). These observations have been validated in independent survival studies (54, 55). There was also an agreement that the presence of rhabdoid or sarcomatoid morphology within any of the morphotypes of renal cell carcinoma represents a form of tumor dedifferentiation. The prognosis of these tumors is similar to that associated with presence of extreme nuclear pleomorphism or tumor giant cells (56, 57). These combined observations were incorporated into a novel grading classification for renal cell carcinoma to be known as ISUP Grading Classification for renal cell carcinoma (Table 1) (58).

**Table 1.** The International Society of Urological Pathology grading classification for renal cell carcinoma (58, 60)

Grade 1	Tumor cell nucleoli invisible or small and basophilic at 400 x magnification
Grade 2	Tumor cell nucleoli conspicuous at 400 x magnification but inconspicuous at 100 x magnification
Grade 3	Tumor cell nucleoli eosinophilic and clearly visible at 100 x magnification
Grade 4	Tumors showing extreme nuclear pleomorphism and/or containing tumor giant cells and/or the presence of any proportion of tumor showing sarcomatoid and/or rhabdoid dedifferentiation

In this classification, grades 1–3 were based on nucleolar prominence, while grade 4 was defined as tumors with highly pleomorphic tumor giant cells or the presence of sarcomatoid and/or rhabdoid morphology. There was consensus that this classification is recommended for papillary and clear cell renal cell carcinoma. It was also agreed that as no current grading system provided independent prognostic information for chromophobe RCC (59). These tumors should not be graded. In addition to its role as a component of grading, it was agreed that sarcomatoid differentiation should be reported separately. As a minimum percentage was not a requirement for diagnostic purposes it was concluded that it was not necessary to report the percentage of sarcomatoid differentiation within individual tumors. Similarly for tumors with rhabdoid morphology, it was agreed that it was unnecessary to report the percentage of rhabdoid tumor present. For both of these dedifferentiation patterns it is, however; necessary to report the underlying primary morphotype. In cases where no primary tumor morphotype is apparent, these should be reported as undifferentiated carcinoma with a sarcomatoid/rhabdoid component.

## Other prognostic factors

In addition to tumor grading, numerous prognostic factors have been investigated for RCC. Prognostic parameters that, according to the consensus conference, should be routinely reported are tumor necrosis and tumor morphotype (22). Tumor necrosis was considered to be of prognostic significance and it was agreed that assessment of this should be based on both macroscopic and microscopic examination. It was recommended that for clear cell RCC, the amount of necrosis should be recorded as a percentage of the sampled tumor. There was agreement that the main tumor morphotypes of RCC were of prognostic significance (60). In particular it was noted that clear cell RCC, stage for stage, has a worse outcome than either chromophobe or papillary RCC. There was also consensus that the subtyping of papillary renal cell carcinoma into types 1 and 2 provides prognostic information. Given that there is no conclusive evidence as to the significance of intra-tumoral microvascular invasion as a prognostic parameter, there was consensus that at present, this should not be considered as a potential staging criterion.

## Conclusion

In keeping with advances in knowledge of renal neoplasia, staging, classification and grading of RCC have undergone major changes in recent times. To reflect this, the ISUP undertook a review of adult renal neoplasia through an international consensus conference in Vancouver in 2012. This review summarizes the guidelines and recommendations from this conference regarding handling/sampling, staging, classification and grading of kidney tumors. It is hoped that such advances in classification will enable pathologists to follow uniformity in reporting of this highly heterogeneous disease.

## References

[1] Siegel R, Naishadham D, Jemal A. (2013). Cancer statistics, CA Cancer J Clin.

[2] Luke C, Sargent N, Pittman K, Price T, Roder D. (2011). Epidemiology of cancers of the kidney in an Australian population.. Asian Pac J Cancer Prev.

[3] Motzer RJ, Porta C, Vogelzang NJ, Sternberg CN, Szczylik C, Zolnierek J, Kollmannsberger C, Rha SY, Bjarnason GA, Melichar B, De Giorgi U, Grünwald V, Davis ID, Lee JL, Esteban E, Urbanowitz G, Cai C, Squires M, Marker M, Shi MM, Escudier B. (2014). Dovitinib versus sorafenib for third-line targeted treatment of patients with metastatic renal cell carcinoma: an open-label, randomised phase 3 trial.. Lancet Oncol.

[4] Hutson TE, Escudier B, Esteban E, Bjarnason GA, Lim HY, Pittman KB, Senico P, Niethammer A, Lu DR, Hariharan S, Motzer RJ. (2014). Randomized phase III trial of temsirolimus versus sorafenib as second-line therapy after sunitinib in patients with metastatic renal cell carcinoma.. J Clin Oncol.

[5] Molina AM, Hutson TE, Larkin J, Gold AM, Wood K, Carter D, Motzer R, Michaelson MD. (2014). A phase 1b clinical trial of the multi-targeted tyrosine kinase inhibitor lenvatinib (E7080) in combination with everolimus for treatment of metastatic renal cell carcinoma (RCC).. Cancer Chemother Pharmacol.

[6] Bonsib SM, Gibson D, Mhoon M, Greene GF. (2000). Renal sinus involvement in renal cell carcinomas.. Am J Surg Pathol.

[7] Bonsib SM. (2004). The renal sinus is the principal invasive pathway: a prospective study of 100 renal cell carcinomas.. Am J Surg Pathol.

[8] Bonsib SM. (2005). T2 clear cell renal cell carcinoma is a rare entity: a study of 120 clear cell renal cell carcinomas.. J Urol.

[9] Thompson RH, Blute ML, Krambeck AE, Lohse CM, Magera JS, Leibovich BC, Kwon ED, Frank I, Cheville JC. (2007). Patients with pT1 renal cell carcinoma who die from disease after nephrectomy may have unrecognized renal sinus fat invasion.. Am J Surg Pathol.

[10] Bonsib SM. (2008). Macroscopic assessment, dissection protocols and histologic sampling strategy for renal cell carcinomas.. Diagn Histopathol.

[11] Sobin LH, Compton CC. (2010). TNM seventh edition: What’s new, what’s changed: communication from the International Union Against Cancer and the American Joint Committee on Cancer.. Cancer.

[12] Edge SB, Byrd DR, Carducci M (2009). AJCC Cancer Staging Manual.

[13] Mostofi FK. (1981). International Histological Classification of Tumors No. 25: Histological Typing of Kidney Tumors.. Geneva cortisone Word Health Organization.

[14] Kovacs G, Akhtar M, Beckwith BJ, Bugert P, Cooper CS, Delahunt B, Eble JN, Fleming S, Ljungberg B, Medeiros LJ, Moch H, Reuter VE, Ritz E, Roos G, Schmidt D, Srigley JR, Störkel S, van den Berg E, Zbar B. (1997). The Heidelberg classification of renal cell tumours.. J Pathol.

[15] Eble JN, Sauter G, Epstein JI, Sesterhenn IA. (2004). Pathology and Genetics of Tumours of the Urinary System and Male Genital Organs.. World Health Organization Classification of Tumours.

[16] Fuhrman SA, Lasky LC, Limas C. (1982). Prognostic significance of morphologic parameters in renal cell carcinoma.. Am J Surg Pathol.

[17] Delahunt B. (2009). Advances and controversies in grading and staging of renal cell carcinoma.. Mod Pathol.

[18] Goldstein NS. (1999). Grading of renal cell carcinoma.. Urol Clin North Am.

[19] Ficarra V, Martignoni G, Maffei N, Brunelli M, Novara G, Zanolla L, Pea M, Artibani W. (2005). Original and reviewed nuclear grade according to the Fuhrman system: a multivariate analysis of 388 patients with conventional renal cell carcinoma.. Cancer.

[20] Delahunt B, Egevad L, Montironi R, Srigley JR. (2013). International Society of Urological Pathology (ISUP) consensus conference on renal neoplasia: rationale and organization.. Am J Surg Pathol.

[21] Srigley JR, Delahunt B, Eble JN, Egevad L, Epstein JI, Grignon D, Hes O, Moch H, Montironi R, Tickoo SK, Zhou M, Argani P. (2013). ISUP Renal Tumor Panel. The International Society of Urological Pathology (ISUP) Vancouver Classification of Renal Neoplasia.. Am J Surg Pathol.

[22] Delahunt B, Cheville JC, Martignoni G, Humphrey PA, Magi-Galluzzi C, McKenney J, Egevad L, Algaba F, Moch H, Grignon DJ, Montironi R, Srigley JR. (2013). Members of the ISUP Renal Tumor Panel. The International Society of Urological Pathology (ISUP) grading system for renal cell carcinoma and other prognostic parameters.. Am J Surg Pathol.

[23] Trpkov K, Grignon DJ, Bonsib SM, Amin MB, Billis A, Lopez-Beltran A, Samaratunga H, Tamboli P, Delahunt B, Egevad L, Montironi R, Srigley JR. (2013). Members of the ISUP Renal Tumor Panel. Handling and staging of renal cell carcinoma: the International Society of Urological Pathology Consensus (ISUP) conference recommendations.. Am J Surg Pathol.

[24] Tan PH, Cheng L, Rioux-Leclercq N, Merino MJ, Netto G, Reuter VE, Shen SS, Grignon DJ, Montironi R, Egevad L, Srigley JR, Delahunt B, Moch H, ISUP Renal Tumor Panel (2013). Renal tumors: diagnostic and prognostic biomarkers.. Am J Surg Pathol.

[25] Fleming S, Griffiths DF. (2005). Best Practice No 180. Nephrectomy for renal tumour; dissection guide and dataset.. J Clin Pathol.

[26] Algaba F, Trias I, Scarpelli M, Boccon-Gibod L, Kirkali Z, Van Poppel H. (2004). Handling and pathology reporting of renal tumor specimens.. Eur Urol.

[27] Mehta V1, Mudaliar K, Ghai R, Quek ML, Milner J, Flanigan RC, Picken MM. (2013). Renal lymph nodes for tumor staging: appraisal of 871 nephrectomies with examination of hilar fat.. Arch Pathol Lab Med.

[28] Che M, Grignon DJ. (2005). Handling and reporting of tumor-containing kidney specimens.. Clin Lab Med.

[29] Axwijk PH, Kluijt I, de Jong D, Gille H, Teertstra J, Horenblas S. (2010). Hereditary causes of kidney tumours.. Eur J Clin Invest.

[30] Verine J, Pluvinage A, Bousquet G, Lehmann-Che J, de Bazelaire C, Soufir N, Mongiat-Artus P. (2010). Hereditary renal cancer syndromes: an update of a systematic review.. Eur Urol.

[31] Shuch B, Singer EA, Bratslavsky G. (2012). The surgical approach to multifocal renal cancers: hereditary syndromes, ipsilateral multifocality, and bilateral tumors.. Urol Clin North Am.

[32] Kuroda N, Tanaka A, Ohe C, Mikami S, Nagashima Y, Sasaki T, Inoue K, Hes O, Michal M, Brunelli M, Martignoni G. (2012). Review of renal oncocytosis (multiple oncocytic lesions) with focus on clinical and pathobiologicalaspects Histol Histopathol..

[33] Tickoo SK, dePeralta-Venturina MN, Harik LR, Worcester HD, Salama ME, Young AN, Moch H, Amin MB. (2006). Spectrum of epithelial neoplasms in end-stage renal disease: an experience from 66 tumor-bearing kidneys with emphasis on histologic patterns distinct from those in sporadic adult renal neoplasia.. Am J Surg Pathol.

[34] Bonsib SM. (2007). Renal veins and venous extension in clear cell renal cell carcinoma.. Mod Pathol.

[35] Zini L, Destrieux-Garnier L, Leroy X, Villers A, Haulon S, Lemaitre L, Koussa M. (2008). Renal vein ostium wall invasion of renal cell carcinoma with an inferior vena cava tumor thrombus: prediction by renal and vena caval vein diameters and prognostic significance.. J Urol.

[36] Thompson RH, Leibovich BC, Cheville JC, Lohse CM, Frank I, Kwon ED, Zincke H, Blute ML. (2005). Should direct ipsilateral adrenal invasion from renal cell carcinoma be classified as pT3a?. J Urol.

[37] Han KR1, Bui MH, Pantuck AJ, Freitas DG, Leibovich BC, Dorey FJ, Zisman A, Janzen NK, Mukouyama H, Figlin RA, Belldegrun AS. (2003). TNM T3a renal cell carcinoma: adrenal gland involvement is not the same as renal fat invasion.. J Urol.

[38] Zhou M, Yang XJ, Lopez JI, Shah RB, Hes O, Shen SS, Li R, Yang Y, Lin F, Elson P, Sercia L, Magi-Galluzzi C, Tubbs R. (2009). Renal tubulocystic carcinoma is closely related to papillary renal cell carcinoma: implications for pathologic classification.. Am J Surg Pathol.

[39] Tickoo SK, dePeralta-Venturina MN, Harik LR, Worcester HD, Salama ME, Young AN, Moch H, Amin MB. (2006). Spectrum of epithelial neoplasms in end-stage renal disease: an experience from 66 tumor-bearing kidneys with emphasis on histologic patterns distinct from those in sporadic adult renal neoplasia.. Am J Surg Pathol.

[40] Aydin H, Chen L, Cheng L, Vaziri S, He H, Ganapathi R, Delahunt B, Magi-Galluzzi C, Zhou M. (2010). Clear cell tubulopapillary renal cell carcinoma: a study of 36 distinctive low-grade epithelial tumors of the kidney.. Am J Surg Pathol.

[41] Argani P, Yonescu R, Morsberger L, Morris K, Netto GJ, Smith N, Gonzalez N, Illei PB, Ladanyi M, Griffin CA. (2012). Molecular confirmation of the t(6;11)(p21;q12) renal cell carcinomas in archival paraffin-embedded material using a break-apart TFEB FISH assay expands its clinciopathologic spectrum.. Am J Surg Pathol.

[42] Merino MJ, Torres-Cabala C, Pinto P, Linehan WM. (2007). The morphologic spectrum of kidney tumors in hereditary leiomyomatosis and renal cell carcinoma (HLRCC) syndrome.. Am J Surg Pathol.

[43] Gill AJ, Pachter NS, Chou A, Young B, Clarkson A, Tucker KM, Winship IM, Earls P, Benn DE, Robinson BG, Fleming S, Clifton-Bligh RJ. (2011). Renal tumors associated with germline SDHB mutations show distinctive morphology.. Am J Surg Pathol.

[44] Debelenko LV, Raimondi SC, Daw N, Shivakumar BR, Huang D, Nelson M, Bridge JA. (2011). Renal cell carcinoma with novel VCL-ALK fusion: new representative of ALK-associated tumor spectrum.. Mod Pathol.

[45] Amin MB1, Gupta R, Ondrej H, McKenney JK, Michal M, Young AN, Paner GP, Junker K, Epstein JI. (2009). Primary thyroid-like follicular carcinoma of the kidney: report of 6 cases of a histologically distinctive adult renal epithelial neoplasm.. Am J Surg Pathol.

[46] Suzigan S, Lopez-Beltran A, Montironi R (2006). Multilocular cystic renal cell carcinoma: a report of 45 cases of a kidney tumor of low malignant potential.. Am J Clin Pathol.

[47] Murakami T, Sano F, Huang Y (2007). Identification and characterization of Birt-Hogg Dube associated renal carcinoma.. J Pathol.

[48] Gobbo S, Eble JN, Delahunt B (2010). Sporadic hybrid oncocytic/chromophobe tumor of the kidney: a clinicopathologic, histomorphologic, immunohistochemical, ultrastructural, and molecular cytogenetic study of 14 cases. Renal cell neoplasms of oncocytosis have distinct morphologic, immunohistochemical, and cytogenetic profile.. Am J Surg Pathol.

[49] Nese N, Martignoni G, Fletcher CD (2011). Pure epithelioid PEComas (so-called epithelioid angiomyolipoma) of the kidney: a clinicopathologic study of 41 cases: detailed assessment of morphology and risk stratification.. Am J Surg Pathol.

[50] Brimo F, Robinson B, Guo C (2010). Renal epithelioid angiomyolipomas with atypia; a series of 40 cases with emphasis on clinicopathologic prognosti c indicators of malignancy.. Am J Surg Pathol.

[51] Delahunt B, Bethwaite PB, Nacey JN. (2007). Outcome prediction for renal cell carcinoma: evaluation of prognostic factors for tumors divided according to histological subtype.. Pathology.

[52] Delahunt B, Srigley JR, Montironi R, Egevad L. (2012). Urological pathology comes of age.. Pathology.

[53] Delahunt B, Sika-Paotonu D, Bethwaite PB (2011). Grading of clear cell renal cell carcinoma should be based upon nucleolar prominence.. Am J Surg Pathol.

[54] Delahunt B, McKenney JK, Lohse CM (2013). A novel grading system for clear cell renal cell carcinoma incorporating tumor necrosis.. Am J Surg Pathol.

[55] Sukov WR, Lohse CM, Leibovich BC (2012). Clinical and pathological features associated with prognosis in patients with renal cell carcinoma.. J Urol.

[56] Delahunt B. (1993). Sarcomatoid renal cell carcinoma: The final common dedifferentiation pathway of renal epithelial malignancies.. Pathology.

[57] Chapman-Fredricks JR, Herrera L, Brancho J (2001). Adult renal cell carcinoma with rhabdoid morphology represents a neoplastic dedifferentiation analagous to sarcomatoid carcinoma.. Ann Diagn Pathol.

[58] Delahunt B, Sika-Paotonu D, Bethwaite PB (2007). Fuhrman grading is not appropriate for chromophobe renal cell carcinoma.. Am J Surg Pathol.

[59] Ficarra V, Martignoni G, Alfano G (2006). Prognostic role of the histologic subtypes of renal cell carcinoma after slide revision.. Eur Urol.

[60] Delahunt B, Srigley JR, Montironi R, Egevad L. International Society of Urological Pathology (ISUP) Grading and Other Prognostic Factors for Renal Neoplasia.. Eur Urol.

